# Identification of Novel *CDH23* Variants Causing Moderate to Profound Progressive Nonsyndromic Hearing Loss

**DOI:** 10.3390/genes11121474

**Published:** 2020-12-09

**Authors:** Khushnooda Ramzan, Nouf S. Al-Numair, Sarah Al-Ageel, Lina Elbaik, Nadia Sakati, Selwa A. F. Al-Hazzaa, Mohammed Al-Owain, Faiqa Imtiaz

**Affiliations:** 1 Department of Genetics, King Faisal Specialist Hospital and Research Centre, P.O. Box 3354, Riyadh 11211, Saudi Arabia; alnumair@kfshrc.edu.sa (N.S.A.-N.); lelbaik@kfshrc.edu.sa (L.E.); fahmad@kfshrc.edu.sa (F.I.); 2 Department of Otolaryngology Head and Neck Surgery, King Faisal Specialist Hospital and Research Centre, P.O. Box 3354, Riyadh 11211, Saudi Arabia; salageel97@kfshrc.edu.sa; 3 Department of Medical Genetics, King Faisal Specialist Hospital and Research Centre, P.O. Box 3354, Riyadh 11211, Saudi Arabia; nsakati@kfshrc.edu.sa (N.S.); alowain@kfshrc.edu.sa (M.A.-O.); 4 College of Medicine, Alfaisal University, Riyadh 11533, Saudi Arabia; salhazzaa@alfaisal.edu

**Keywords:** nonsyndromic hearing loss, *DFNB12*, *CDH23*, whole exome sequencing, missense variants, phenotypic variability, Saudi Arabia

## Abstract

Mutant alleles of *CDH23*, a gene that encodes a putative calcium-dependent cell-adhesion glycoprotein with multiple cadherin-like domains, are responsible for both recessive *DFNB12* nonsyndromic hearing loss (NSHL) and Usher syndrome 1D (*USH1D*). The encoded protein cadherin 23 (CDH23) plays a vital role in maintaining normal cochlear and retinal function. The present study’s objective was to elucidate the role of *DFNB12* allelic variants of *CDH23* in Saudi Arabian patients. Four affected offspring of a consanguineous family with autosomal recessive moderate to profound NSHL without any vestibular or retinal dysfunction were investigated for molecular exploration of genes implicated in hearing impairment. Parallel to this study, we illustrate some possible pitfalls that resulted from unexpected allelic heterogeneity during homozygosity mapping due to identifying a shared homozygous region unrelated to the disease locus. Compound heterozygous missense variants (p.(Asp918Asn); p.(Val1670Asp)) in *CDH23* were identified in affected patients by exome sequencing. Both the identified missense variants resulted in a substitution of the conserved residues and evaluation by multiple in silico tools predicted their pathogenicity and variable disruption of CDH23 domains. Three-dimensional structure analysis of human CDH23 confirmed that the residue Asp918 is located at a highly conserved DXD peptide motif and is directly involved in “Ca^2+^” ion contact. In conclusion, our study identifies pathogenic *CDH23* variants responsible for isolated moderate to profound NSHL in Saudi patients and further highlights the associated phenotypic variability with a genotypic hierarchy of *CDH23* mutations. The current investigation also supports the application of molecular testing in the clinical diagnosis and genetic counseling of hearing loss.

## 1. Introduction

Hearing loss (HL), an etiologically heterogeneous trait, is the most frequent sensory impairment affecting 1–3 out of every 1000 children at birth or during early childhood [1,2]. HL can be caused by genetic or environmental factors, due to an association between these factors, and has major clinical, social, and quality of life implications. Approximately more than 50% of all congenital cases are hereditary, with nonsyndromic hearing loss (NSHL) being the most common, accounting for 75% of all the cases [2,3]. NSHL is often sensorineural and can be transmitted as autosomal recessive (*DFNB*, 80%), autosomal dominant (*DFNA*, 15–20%), and X-linked trait (*DFN*, 1%), or by a mitochondrial pattern of inheritance (<1%) [4,5]. To date, a total of 170 loci and 115 genes responsible for NSHL have been identified (Hereditary Hearing Loss Homepage; Appendix A).

Recessive mutations of the *CDH23* gene (MIM#605516) are responsible for both nonsyndromic deafness 12 (*DFNB12*, MIM#601386) and Usher syndrome type 1D (*USH1D*, MIM#601067) [6,7,8]. *DFNB12* is characterized by prelingual-onset sensorineural NSHL, without the impairment of visual or vestibular functions. Conversely, individuals with *USH1D* are associated with severe manifestations, including congenital severe to profound deafness, variable vestibular areflexia, and progressive adolescent-onset vision loss due to retinitis pigmentosa (RP) [9,10,11].

The significance of *CDH23* as a deafness gene and the associated phenotypic spectrum of *CDH23* mutations has been widely studied among different ethnic populations, and an interesting genotype-phenotype correlation is suggested based on the pathogenic potential of the variants (The Human Gene Mutation Database, HGMD). Missense *CDH23* variants usually underlie a milder phenotype of NSHL, known as *DFNB12*. In contrast, protein-truncating *CDH23* mutations due to frameshift, splice site, or nonsense pathogenic variants are causative of the severe phenotype of Usher syndrome [8,9,12]. The encoded protein, cadherin 23 (CDH23), belongs to the cadherin superfamily, which constitutes a family of transmembrane proteins that mediate calcium-dependent cell-cell adhesion. CDH23 has essential roles in establishing and maintaining the proper organization of the stereocilia bundle of hair cells in the cochlea and vestibule during late embryonic and early postnatal development. It is a part of the functional network formed by CDH23, MYO7A, USH1C, and USH1G, which regulates hair bundle morphogenesis and is essential for proper mechanotransduction in hair bundles of the inner-ear neurosensory cells [13].

We describe a consanguineous Saudi family in which four siblings had moderate to severe high-frequency progressive NSHL, without any vestibular or ocular involvement. Detailed clinical and molecular genetic analyses were performed. Autozygosity mapping followed by whole-genome SNP genotyping, failed to identify any possible block of homozygosity encompassing a known NSHL gene. Whole exome sequencing (WES) was further used to identify compound heterozygous *CDH23* variants as the probable genetic cause of the *DFNB12* phenotype in this family. Moreover, the effects of the identified variants on protein structure were assessed, and we discuss the pathogenic potential and clinical fate of the identified *CDH23* variants.

## 2. Materials and Methods

### 2.1. Study Subjects and Ethical Considerations

**Figure 1:**
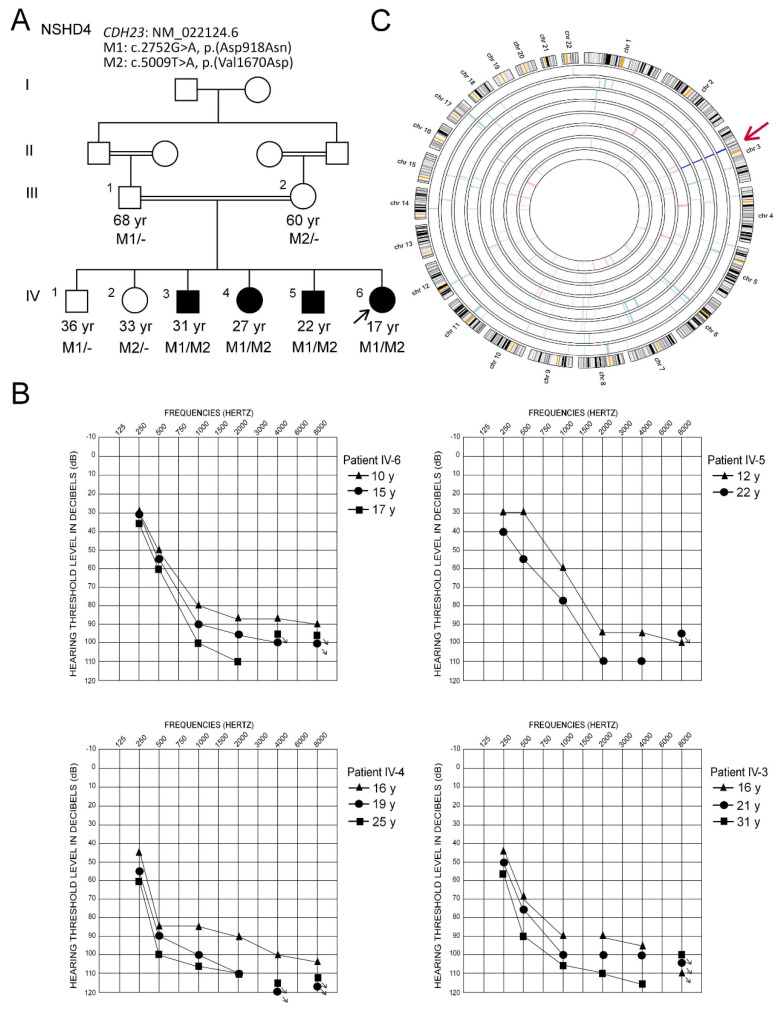
(**A**) Pedigree of the study family (NSHD4), segregating nonsyndromic hearing loss. Circles and squares denote females and males, respectively (solid symbols indicate affected individuals). Genotypes for the two identified mutations in *CDH23* are shown below the symbols of each tested family member. *CDH23*: M1/- or M2/- indicate heterozygous carriers of the c.2752G>A, p.(Asp918Asn) and c.5009T>A, p.(Val1670Asp), respectively. *CDH23*: M1/M2 indicates compound heterozygous individuals. (**B**) Representative pure-tone audiometric results in the best ear of the patients. Hearing-impaired family members illustrate mild sloping to profound hearing loss for the younger siblings (IV-5 and IV-6) and moderate sloping to profound hearing loss for older siblings (IV-3 and IV-4). The affected patients showed progressive nature of HL, as confirmed by audiograms obtained at different ages. (**C**) AgileMultiIdeogram output of autozygosity analysis showing a single common region of homozygosity (ROH) between the four affected members of the family (IV-3, IV-4, IV-5, IV-6, dark blue) on chromosome 3, which is not shared with any of the unaffected individuals (III-1, III-2, IV-1, IV-2, pink). The shared ROH is indicated by a red arrow.

A Saudi family (NSHD4; Figure 1A) was referred to the Department of Medical Genetics at King Faisal Specialist Hospital and Research Centre (KFSH&RC), Riyadh, Saudi Arabia, for molecular exploration of genes implicated in HL. The family consists of four siblings presenting with HL (IV-3, IV-4, IV-5, IV-6), two unaffected siblings (IV-1, IV-2), and healthy first cousin parents (III-1, III-2). Family information to draw the pedigree was obtained by interviewing the parents (Figure 1A). The study was approved by the institutional review board (RAC#2100001). Written informed consent was obtained from all the participating individuals. The experimental procedures were carried out in the First Arabian Hereditary Deafness (FAHD) Unit of KFSH&RC following the Declaration of Helsinki.

### 2.2. Clinical Evaluation of Subjects

The affected siblings were thoroughly examined in the Department of Otolaryngology at KFSH&RC. Detailed medical histories and physical examinations were carried out to exclude any possible environmental causes or syndromic forms of HL. Pure-tone audiometry on affected individuals was performed at frequencies between 250 and 8000 Hz in a sound-treated room, following current clinical standards. The severity of hearing loss was defined as mild (26–40 dB), moderate (41–60 dB), severe (61–80 dB), or profound (>81 dB). A computerized tomography (CT) scan of the temporal bone was obtained to look for inner ear anomalies. The vestibular function was evaluated via tandem gait and Romberg testing. The ophthalmological evaluation included the eye fundus and visual field examination.

### 2.3. DNA Extraction and Whole-Genome SNP Genotyping Using Axiom^TM^ 6.0 Array

Blood samples were obtained from the four affected individuals and their family members. The genomic DNA was extracted using Gentra Puregene Blood kit (Qiagen, Germantown, MD, USA). The integrity and quantity of the extracted DNA samples were assessed through NanoDrop spectrophotometer (Thermo Scientific, Wilmington, DE, USA). DNA of both the affected and unaffected individuals was subjected to genome-wide single nucleotide polymorphism (SNP) genotyping, using Axiom^TM^ CEU Human Array 6.0 and Gene Titan MC Instrument (Affymetrix Inc., Santa Clara, CA, USA), which provide high genetic coverage of 587,352 SNPs across the whole genome. Analyses for annotated regions of the absence of heterozygosity (AOH) for each sample and shared runs of homozygosity (ROH) in the affected cases were performed using AgileMultiIdeogram.

### 2.4. Whole Exome Sequencing (WES), Data Processing, and Variant Analysis

DNA samples from two affected individuals (IV-5 and IV-6; Figure 1A) underwent exome sequencing. The exonic library preparation was performed using the Agilent SureSelectXT human all exon platform, which provides high end-to-end coverage of the complete coding regions of the genome. In short, after initial sample quality control, 50 ng of DNA was fragmented using QXT enzymatic protocol followed by adaptor tagging (Agilent Technologies, Santa Clara, CA, USA). Paired-end sequencing was performed on a HiSeq2000 instrument (Illumina Inc., San Diego, CA, USA) according to the manufacture’s protocol. The raw data were evaluated for read quality with FastQC software (Babraham Bioinformatics). After removing low-quality reads, Burrows–Wheeler Aligner [14] and SAMTOOLS [15] were used to align sequences, copy number variants (CNVs), and small indels, to the UCSC Human Genome Database (UCSC, GRCh37/hg19). Variants were called using the Genome Analysis Toolkit (GATK; The Broad Institute, Cambridge, MA, USA) [16], and the variant annotation and filtering were performed using ANNOVAR [17]. A web-based tool, VCF2CNA, was used to detect CNVs in the Variant Call Format (VCF) files. Minor allele frequency (MAF) of the variants was determined using publicly available variant databases: dbSNP147, 1000Genomes (NCBI browser), ExAC and gnomAD (The Broad Institute). Furthermore, the variants that are frequent in our in-house Saudi Human Genome Program (SHGP) database, which is based on >3000 Saudi individuals, were also filtered out.

### 2.5. Sanger Validation and Segregation Analysis

Bidirectional sequencing was carried out to verify the variants of interest identified by WES. Primers flanking candidate variants were designed using Primer3 software. The amplified PCR products were sequenced using the BigDye Terminator v3.1 Cycle Sequencing kit and an ABI3130xl sequencer (Applied Biosystems, Foster City, CA, USA). Sequencing data were examined with the SeqManII module of Lasergene (DNA Star Inc., Madison, WI, USA) software. All family members were screened for the filtered candidate variants to establish their segregation with the disease phenotype.

### 2.6. Pathogenicity Computation and In Silico Modeling

Possible pathogenic effects of the missense single-nucleotide variants (SNVs) on CDH23 protein function were evaluated using multiple pathogenicity-computation tools, including PolyPhen-2, SIFT, DANN, MutationTaster, FATHMM-MKL, MetaSVM, MetaLR, MutationAssessor, and CADD. Clustal Omega and the Genomic Evolutionary Rate Profiling (GERP++) algorithm were used to estimate the conservation of mutated residues (Asp918 and Val1670).

To predict the impact of both the identified missense variants, located in the protein domains cadherin 9 and cadherin 16, CDH23 sequences were obtained from the Uniprot database (Q9H251). Homology models for both the domains affected by the SNVs were predicted by performing template search with BLASTp [18] and HHBlits [19] against the SWISS-MODEL template library (SMTL version 2019-10-02) utilizing the solved molecular models in the Protein Data Bank (PDB). The best structure templates matching the target sequences were selected for each domain: cadherin 9 (3q2w.1.A) and cadherin 16 (5wjm.1.A). ProMod3 was used to build models based on the target–template alignment [20]. The conserved coordinates between the template and the target were copied to the model. Global and per-residue model qualities were estimated using the QMEAN scoring function [21]. The molecular graphics program PyMOL was used to visualize the model, examine the consequences of introducing the mutations, p.(Asp918Asn) and p.(Val1670Asp), and produce figures. Mutagenesis tool within PyMOL was used to mutate the native residue and the native side chain was then substituted by the “best” rotamer of the mutant amino acid, which totalizes the lowest score based on the lowest energy (least collisions and most favorable hydrogen bonds).

## 3. Results

### 3.1. Clinical Description and Hearing Characteristics

A consanguineous Saudi family (NSHD4) presented with hearing loss in four of their children (Figure 1A). The ages for the patients were 17 to 31 years at the time of the study. A thorough clinical examination of the patients showed a mild sloping to profound sensorineural HL for the younger two siblings (IV-5 and IV-6) and a moderate sloping to profound sensorineural HL for the two older siblings (IV-3 and IV-4). The affected patients showed a progressive nature of HL, as confirmed by audiograms obtained at different ages (Figure 1B). All four patients are using binaural hearing aids with significant benefits. They were able to identify words in a close-set with no visual cues at normal conversation levels. However, they rely on lip-reading for communication. Their aided scores with current hearing aids were in the range of mild to moderate HL (thresholds between 30 and 55 dB HL), suggesting significant functional gain with amplification. CT of the temporal bone revealed that both mastoids were normally pneumatized. The inner ear structures, including the left and right cochlear, vestibules, semicircular canals, and middle ear structures, were normal. No gross vestibular dysfunction was reported by any of the patients, and there was no delay in the motor milestones. The patients did not display RP or any other ophthalmological manifestations. There were no extended family members with congenital or progressive HL. There was no history of head trauma, exposure to ototoxic noise levels, aminoglycoside antibiotics, or systemic or otic infections that might underlie the HL in the four affected siblings. These findings suggested the possibility of genetic involvement underlying NSHL in the main family, and the DNA samples from all available family individuals were used for genetic analysis.

### 3.2. Genetic Analysis

#### 3.2.1. Autozygome Analysis

SNP genotypes were analyzed using AgileMultiIdeogram to determine regions of AOH and common ROHs in affected four siblings (V-3, V-4, V-5, and V-6; Figure 1A). A single shared region of homozygosity at chromosome 3q13.2–11q23.2 (chr3: 65,010,372–68,988,334 bp; UCSC hg19) was identified (Figure 1C). This region corresponded to a 3.97-Mb region on the human Genome Data Viewer (annotation release 109), which contained seven labeled genes. The affected individuals were products of a consanguineous union, and as presumed from the pedigree, the possibility of homozygous mutation was more likely, but this region did not contain any known or potential HL gene; therefore, we next proceeded with exome sequencing.

#### 3.2.2. Identification of Mutations by Whole Exome Sequencing

Exome data of two affected individuals (IV-5 and IV-6; Figure 1A) were obtained from the HiSeq 2000 platform (Illumina Inc., San Diego, CA, USA), with an average sequence depth of on-target regions of 97× for affected individual IV-5 and 106× for IV-6. The average percentage of bases in the target region was 97.8% and 93.2%, with a 10× and 20× coverage, respectively. Variants with a quality score (QUAL) of ≤30 (Q30) were selected only. For CNV detection, a VCF file containing paired patient and normal control individual data was uploaded and analyzed by the VCF2CNA algorithm using the standard parameters. A CNV profile of the patient IV-5 was obtained containing 453 CNVs, overlapping at least one coding exon. These CNVs were also present in the normal control individuals from the same ethnic population; therefore, they were not considered further.

**Figure 2:**
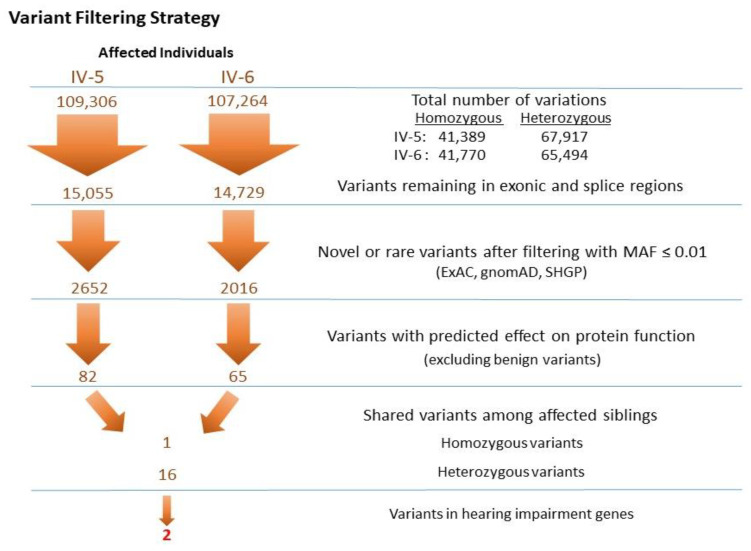
Whole exome sequencing and variant filtering strategy adapted to narrow down the most promising causative mutations in Family NSHD4. The exome was interrogated for variants present in genes shared by two affected individuals under an autosomal recessive model.

The VCF files of the two affected individuals were annotated and filtered using Illumina Variant Studio software to ascertain potentially damaging variants underlying the HL phenotype. A workflow for variant filtering scheme of exome data used for variants prioritization and following the genetic analysis is illustrated in Figure 2. In short, the variants’ genomic positions were taken into account, and the variants located in intergenic, intronic, and untranslated regions (UTRs) were excluded. For individual IV-5, a total of 109,306 variants (41,389 homozygous and 67,917 heterozygous) and for individual IV-6, 107,264 variants (41,770 homozygous and 65,494 heterozygous) were identified. Based on family history and pedigree, an autosomal recessive mode of inheritance was considered, and homozygous or compound heterozygous variants were anticipated. The variants from WES were filtered such that: coding/splicing variants, novel or variants with MAF below 0.01% in 1000Genomes, ExAC and gnomAD, variants not frequently observed in our in-house Saudi exomes database, variants that are predicted to be likely pathogenic/pathogenic, and shared among the two affected siblings, were considered as likely causal variants. A list of last filtered variants shared among the exome data of two affected siblings is in Table S1. The indels detected in the exome data were filtered out in the variant filtration strategy and were not prioritized further. MultiIdeogram analysis for regions of AOH for each affected sample and the few shared ROHs among the siblings’ pair (Figure 1C) could explain a shortlist of shared homozygous variants in the exome data of the affected siblings IV-5 and IV-6. The in-house exome database has also led us to more filtration power and made this list even shorter.

**Figure 3:**
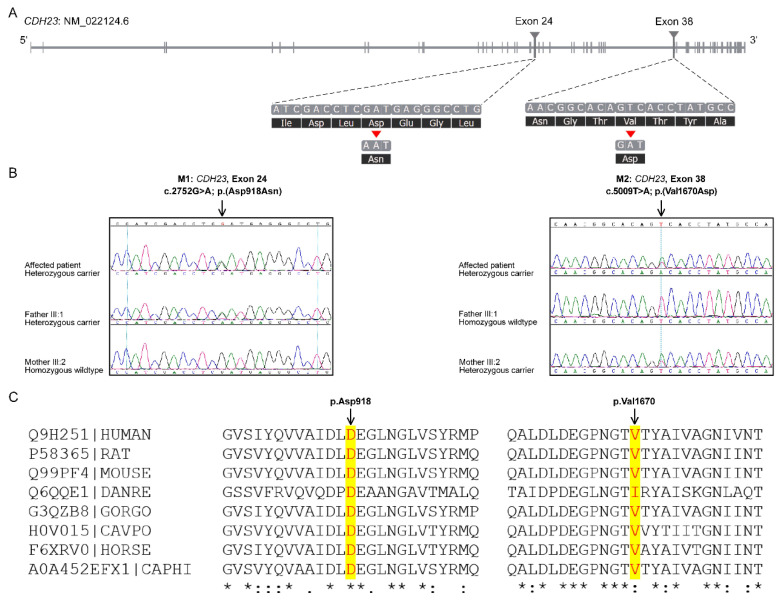
Identification of *CDH23* (NM_022124.6) mutations by whole exome sequencing. (**A**) The schematic of the *CDH23* gene that is 290 kb in length. The triangles locate the positions of the two heterozygous mutations identified in exons 24 and 38 by exome sequencing. (**B**) Electropherogram profiles of the index patient and unaffected parents showing the inheritance of the missense mutations, (c.2752G>A, p.(Asp918Asn) and c.5009T>A, p.(Val1670Asp)). (**C**) Conservation of Asp918 and Val1670 amino acids is observed across species (highlighted in yellow). Asterisk (*) indicates positions which have a single, fully conserved residue. Colon (:) indicates conservation between groups of strongly similar properties-scoring > 0.5.

In short, the variants identified in any gene associated with HL phenotype were carefully prioritized by allele frequency and predicted molecular phenotypic effect. According to the clinical phenotypes and autosomal recessive inheritance patterns combined with the database analysis, two variants of the *CDH23* gene (NM_022124.6); c.2752G>A, p.(Asp918Asn) in exon 24, and c.5009T>A, p.(Val1670Asp) in exon 38, were considered as likely pathogenic compound heterozygous variants causing the disease presentation (Figure 3A). The identified genetic variants were validated by Sanger sequencing (Figure 3B). Genotyping in the parents confirmed that these mutations co-segregated with deafness within the family; the variant c.2752G>A was inherited from the father (III-1), while c.5009T>A was inherited from the mother (III-2) (Figure 3B). The unaffected siblings (IV-1 and IV-2) were also carriers (Figure 1A).

#### 3.2.3. Prediction of the Pathogenic Significance of the Mutations

The pathogenicity scores of CDH23 SNVs by multiple in silico bioinformatics tools predicted the variants to be damaging for the protein function (Table 1). The p.(Asp918Asn) and p.(Val1670Asp) variants have a CADD score of 28.3 and 24.5, respectively. Upon sequence comparison across various species, a high degree of evolutionary conservation was observed for both the amino acid residues at the mutation sites (Figure 3C). The GERP++ score of 5.4 and 5.76 was obtained for Asp918 and Val1670, respectively (Table 1). The p.(Asp918Asn) has already been reported as a causative *CDH23* mutation in HL patient (HGMD: CM140352 [22]), while the p.(Val1670Asp) is a novel variant.

#### 3.2.4. Impact of p.(Asp918Asn) and p.(Val1670Asp) Mutations on the CDH23 3D Structure

**Figure 4:**
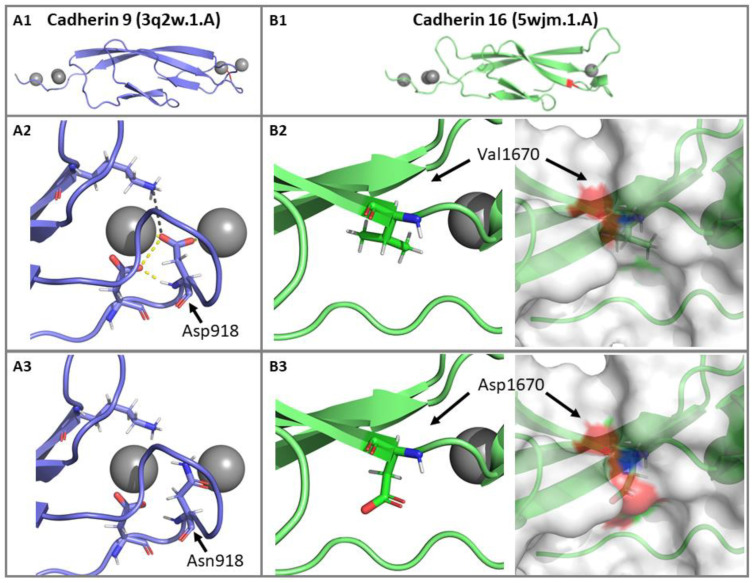
The generated 3D homology model of the CDH23 protein. The Asp918 and Val1670 mutation sites are located at extracellular cadherin domains EC9 and EC16, respectively. CDH23 sequences were obtained from the Uniprot database (Q9H251), and the best structure templates matching the target sequences were selected for each domain (**A1**) cadherin 9 (3q2w.1.A) and (**B1**) cadherin 16 (5wjm.1.A). (**A2**) A close-up view of the wild-type residue p.Asp918 at the mutation site, with the hydrogen bond interaction shown as a yellow dashed line. Asp918 amino acid is part of a highly conserved peptide motif DXD and is directly involved in “Ca^2+^” ion contact required for the interdomain rigidification of the cadherin repeat domains. (**A3**) The introduction of Asn residue at position 918 would abrogate the “Ca^2+^” ion contact and other bonds, thereby disrupting the structure of the Cadherin 9 domain. (**B2**) A close-up view of the wild-type residue p.Val1670 at the mutation site. (**B3**) The bigger negatively charged mutant residue Asp at position 1670 results in loss of hydrophobic interactions causing a clash or repulsion with the neighboring residues, thus affecting the stability of the domain. Homology models confirm the variable disruption of structurally and functionally critical CDH23 domains.

The identified variant p.(Asp918Asn) is located in the Cadherin 9 domain of the CDH23 protein. Analysis of the homology model of the crystal structure of human CDH23 showed that Asp918 is directly involved in a metal “Ca^2+^” ion contact, H-bond with aspartic acid at position 5, and a salt bridge with lysine 64. The mutant residue “Asn” is neutral compared to negatively charged wild-type residue “Asp”. In the p.(Asp918Asn) mutant structure, the difference in charge disturbs the ionic interactions made by the wild-type residue. Notably, loss of negative charge (oxygen atoms of the Asp “−COO−”) results in the loss of binding with the Ca^2+^, thus disturbing the domain (Figure 4A). The second variant p.(Val1670Asp) is located within the Cadherin 16 domain on the protein’s surface; replacement of the native amino acid with a negatively charged mutant residue results in loss of hydrophobic interactions with other molecules or other parts of the protein. The bigger-sized mutant residue may also cause a clash and repulsion with the neighboring residues, affecting the domain’s stability (Figure 4B). CDH23 homology models confirm the variable disruption of both structurally and functionally critical CDH23 domains, which may impact the normal development of hearing.

## 4. Discussion

Autosomal recessive NSHL (arNSHL), one of the most frequent genetic disorders in humans, is subjected to extensive genetic heterogeneity, therefore rendering molecular diagnosis difficult. Homozygosity mapping in consanguineous families provides a means to detect genes causing recessive Mendelian disorders by identifying chromosomal regions with shared ROH among the affected family members [23,24,25,26]. Although it is much more probable for a related spouse to carry the same recessive mutation than a different mutation, compound heterozygosity can still occur in the setting of consanguinity [27,28]. In the study family (NSHD4) with four affected siblings with arNSHL, initial autozygosity analysis suggested a novel locus, but none of the genes within the autozygome explained the HL phenotype. The potential pitfalls that arose during the course of homozygosity mapping of the family’s NSHL gene resulted from unexpected allelic heterogeneity and identification of a shared ROH region unrelated to the disease locus. Consistent with the recessive inheritance of this family, we later identified compound heterozygous *CDH23* mutations by successfully and efficiently applying WES for the molecular diagnosis of arNSHL in all four siblings. With the more recent availability of next-generation sequencing (NGS) technologies, pathogenic variant identification, especially in highly heterogeneous disorders including hearing impairment phenotype, has been significantly improved by obviating the prerequisite to prioritize genes for sequencing within candidate autozygous loci.

The *CDH23*, located on chromosome 10q21-q22, encompasses more than 290 kb and consists of 70 exons. The gene encodes a 3354 amino acid protein with 27 extracellular cadherin (EC) repeat domains (exons 2–64), a single transmembrane domain (exon 65), and a short cytoplasmic domain (exons 66–70). The encoded protein, cadherin 23 (CDH23), is among the 113 human cadherin superfamily members, which constitutes a family of integral transmembrane proteins that mediate Ca^2+^-dependent cell-cell adhesion. CDH23 is involved in the establishment of cell-cell contacts and the organization of the EC matrix. The EC domains interact with other cadherin molecules in cis and trans to form homo-dimeric interactions, which are essential to mechanically hold the opposing cell surfaces together. This stability is achieved by binding of Ca^2+^ ions to highly conserved cadherin-specific amino acids motifs such as LDRE, DXD, and DXNDN located in each EC domain. These conserved peptide sequences are required for Ca^2+^ binding, linearization, rigidification, and dimerization of the cadherin molecules [29,30]. Protein structure analyses suggest that mutations in such domains perturb CDH23 dimerization and might impair the interactions, change the local surface structure, and destabilize CDH23 protein structure affecting the function of the protein [31]. Both p.(Asp918Asn) and p.(Val1670Asp) mutations identified in our patients involved conserved CDH23 amino acids (Figure 3C) and were predicted to have severe detrimental effects by multiple in silico tools (Table 1). The altered protein conformations indicated that both the wild-type residues are indispensable for the protein function of CDH23 (Figure 4). The mutation p.(Asp918Asn) disrupts the highly conserved peptide motif DXD at cadherin domain 9, where Asp918 is directly involved in binding to the Ca^2+^ ions for the interdomain rigidification of the cadherin repeat domains. Biallelic pathogenic mutations at position 918 have been previously associated with arNSHL; c.2752G>A, p.(Asp918Asn) mutation was identified in an Indian family [22] and c.2752G>C, p.(Asp918His) in Chinese Hans [32]. Based on the American College of Medical Genetics (ACMG) guidelines for variant classification [33], variant p.(Asp918Asn) was classified as pathogenic (PM1, PM2, PP1, PP2, PP3), and p.(Val1670Asp) as a likely pathogenic variant (PM2, PP1, PP2, PP3). The novel *CDH23* variant p.(Val1670Asp) is deposited in the Leiden Open Variation Database (LOVD#00314956) and ClinVar (accession#SUB8608268). The variants are also registered as mutations in the Saudi Human Genome Program database.

The allelic disorders *DFNB12* and *USH1D* were first mapped to the long arm of chromosome 10 [34,35], and the causative mutations in *CDH23* were subsequently identified [7,8]. The mutation spectrum of *CDH23* phenotypes is diverse, and a total of 400 different mutations in this gene have been described to date with an interesting genotype-phenotype correlation [9,12,36]. *CDH23* mutations can cause distinct disease outcomes. The patients with recessive *CDH23* variants display a wide range of hearing and vision loss phenotypes differing in severity, age at onset, and presence or absence of vestibular areflexia. The majority of *CDH23* mutations have been associated with congenital or prelingual-onset, severe-to-profound sensorineural HL either as nonsyndromic *DFNB12* or syndromic *USH1D* [6,37,38]. Individuals with NSHL usually carry *CDH23* missense mutations, which are assumed to be hypomorphic alleles with sufficient residual activity for retinal and vestibular function, but not sufficient for the auditory cochlear function, thereby causing hearing loss *DFNB12* phenotype. On the contrary, *CDH23* null alleles (due to frameshift, splice-site, or nonsense variants), and some missense *CDH23* mutations cause deafness/blindness syndrome, *USH1D* [6,8]. It was further hypothesized that *USH1D* occurs only in the presence of two *USH1D* alleles in trans, while a *DFNB12* allele in trans with a *USH1D* allele results in *DFNB12* phenotype, suggesting the phenotypically dominant nature of a *DFNB12* allele, which can preserve the normal retinal and vestibular function even in the presence of a *USH1D* allele [38].

HL progression is also reported as an essential clinical feature caused by *CDH23* mutations. The contribution of *CDH23* to adult-onset postlingual progressive sensorineural HL in Caucasians, Japanese and Korean adults has been documented [38,39,40]. Four affected siblings with progressive hearing impairment in our study carried a known pathogenic missense *CDH23* variant, previously known as the prelingual *DFNB12* variant, in a trans configuration with another rare novel *CDH23* missense variant. Taken together, both the detected variants were missense mutations, thereby corroborating the previous reports regarding the *DFNB12* phenotype, as the affected individuals in our family had isolated sensorineural NSHL with no extra-audiological features.

CDH23 is expressed in various structures within the inner ear: the utricular–saccular foramen, ductus reuniens, Reissner’s membrane, and particularly in sensory inner and outer hair cells, where it is a component of the tip-links in the hair cell stereocilia [41,42,43]. CDH23 is supposed to be critical for the crosslinking of the stereocilia. CDH23 co-localizes with protocadherin-15 (PCDH15) and both are localized in the upper and lower parts of the tip-link complex, respectively. CDH23 homodimers interact in trans with PCDH15 homodimers to form tip-link filaments, and they play a crucial role in mechanoelectrical transduction channels in the hair bundles of cochlear hair cells [42,44]. Mutations in *CDH23* or *PCDH15* that affect their interaction severely disrupt hair-bundle morphology, causing sensory impairment [45]. The murine ortholog, which carries a *Cdh23* null allele, gives rise to waltzer phenotype and leads to disorganized, splayed stereocilia, and mimic *USH1D* exhibiting deafness and vestibular dysfunction [46,47]. While in contrast, the salsa mice suffer from progressive HL due to a *Cdh23* missense mutation that is predicted to disturb Ca^2+^ binding by the EC CDH23 domain, modeling the *DFNB12* phenotype. Unlike the mice with *Cdh23* null alleles, hair cell development in salsa mice with a *Cdh23* missense mutation is unaffected. Instead, tip-links were found to be progressively lost, resulting in hair cell death, suggesting that similar mutations in *DFNB12* patients lead to HL by affecting the tip links [48]. Moreover, *Cdh23^ahl^* mutant alleles in mice have been linked to age-related progressive HL with varying degrees of progression, as explained by allelism and modifier genes [49,50]. A strain-specific *Cdh23* is also implicated in noise-induced HL susceptibility [47,51,52]. The *CDH23* variants in human subjects have been associated with age-related and noise-induced HL; however, its relative contribution has not been adequately investigated [39,40,53]. The underlying mechanisms responsible for different phenotypes of *CDH23* mutations have not yet been thoroughly elucidated.

## 5. Conclusions

In conclusion, our analyses identified compound heterozygous mutations in the *CDH23* gene associated with congenital high-frequency recessively inherited hearing loss phenotype. Our findings add to the mutation spectrum of the *CDH23*, explain the phenotypic variability associated with *CDH23* mutant alleles, and further provide a basis for the genotypic hierarchy of *CDH23* mutations, depending on the pathogenic potential of the variants. Moreover, we support the application of NGS in the early diagnosis of HL, effective rehabilitation, and its implications for informed genetic counseling.

## Figures and Tables

**Table 1:** *CDH23* variants associated with nonsyndromic hearing loss identified in family NSHD4.

**Genomic** **Coordinates** **and nomenclature**		***CDH23* (10q22.1)**	
		**Variant 1**	**Variant 2**
	Genomic Position (hg19/GRCh37)	chr10:73464686	chr10:73537600
	dbSNP ID	rs769870573	rs397517333
	HGVS RefSeq:NM_022124.6	exon 24 of 70 c.2752G>A, p.(Asp918Asn)	exon 38 of 70 c.5009T>A, p.(Val1670Asp)
**Global minor allele frequency (MAF)**	GnomAD_exomes	A = 0.000008 (2/246770)	A = 0.000068 (17/249302)
	ExAC	A = 0.000017 (2/117490)	A = 0.000058 (7/120554)
	SHGP	0	0
**In silico pathogenicity prediction tool**	CADD Score ^a^	Pathogenic (28.3)	Pathogenic (24.5)
	PolyPhen-2	Probably damaging (0.999)	Possibly damaging (0.735)
	SIFT	Deleterious (0)	Deleterious (0)
	MutationTaster	Disease causing (1)	Disease causing (0.9954)
	Mutation assessor	High (3.985)	High (3.82)
	DANN	Pathogenic (0.9991)	Pathogenic (0.9807)
	FATHMM-MKL	Damaging (0.9939)	Damaging (0.9695)
	MetaSVM	Damaging (0.3538)	Damaging (0.5357)
	MetaLR	Damaging (0.5892)	Damaging (0.6209)
	Conservation GERP++ ^b^	Conserved (5.4)	Conserved (5.76)
**ACMG variant classification** ^c^		PM1, PM2, PP1, PP2, PP3	PM2, PP1, PP2, PP3
		“Pathogenic”	“Likely Pathogenic”

^a^ CADD scores are derived from several different functional annotation tools. A score of 20 indicates that a variant is amongst the top 1% of deleterious variants in the human genome. The higher the score, the more likely that variant is predicted to be damaging to the protein. ^b^ Genomic Evolutionary Rate Profiling (GERP) is a conservation score calculated by quantifying substitution deficits across multiple alignments of orthologues using the genomes of 35 mammals. It ranges from −12.3 to 6.17, with 6.17 being the most conserved. ^c^ Classified based on the American College of Medical Genetics (ACMG) guidelines.

## Supplementary Materials

The following are available online at https://www.mdpi.com/2073-4425/11/12/1474/s1, Table S1: contains list of shared variants among the two affected siblings (IV-5 and IV-6, Figure 1A) after strategic filtration as depicted in Figure 2, with the *CDH23* pathogenic variants highlighted.

## Appendix A. Web Resources

1000Genome	http://browser.1000genomes.org/
ANNOVAR	http://annovar.openbioinformatics.org/
Burrows-Wheeler Aligner (BWA) aligner	(http://bio-bwa.sourceforge.net/)
ClustalW2 for Multiple Sequence Alignment	http://www.ebi.ac.uk/Tools/msa/clustalw2/
Combined Annotation Dependent Depletion (CADD)	https://cadd.gs.washington.edu/
dbSNP	http://www.ncbi.nlm.nih.gov/snp/
ExAC	http://exac.broadinstitute.org/
FATHMM-MKL	http://fathmm.biocompute.org.uk/fathmmMKL.htm
Genome Aggregation Database (gnomAD)	http://gnomad.broadinstitute.org/
Genomic evolutionary rate profiling (GERP)	http://mendel.stanford.edu/SidowLab/downloads/gerp/
Hereditary hearing loss homepage	http://hereditaryhearingloss.org/
Human gene mutation database	http://www.hgmd.cf.ac.uk/ac/
Human genome data viewer	https://www.ncbi.nlm.nih.gov/genome/gdv/
Leiden open variation database (LOVD)	http://databases.lovd.nl/shared/genes/CDH23)
MutationAssessor	http://mutationassessor.org/r3/
MutationTaster	http://www.mutationtaster.org/
National Center for Biotechnology Information (NCBI)	http://www.ncbi.nlm.nih.gov/
Online Mendelian inheritance of man (OMIM)	https://www.omim.org/
Polyphen-2 (Polymorphism Phenotyping)	http://genetics.bwh.harvard.edu/pph2/
Primer3web (v4.1.0)	https://primer3.ut.ee/
Protein Data Bank (PDB)	http://www.wwpdb.org/
PyMOL Molecular Graphics System, Schrodinger, LLC	https://pymol.org/
SAMTOOLS	http://samtools.sourceforge.net/
Saudi Human Genome Program (SHGP)	https://shgp.sa/
SIFT (Sorting Intolerant from Tolerant)	http://sift.jcvi.org/
UCSC Human Genome Database	http://www.genome.ucsc.edu/
VCF2CNA	http://vcf2cna.stjude.org/
