# Supplementary material for: Identification of Novel CDH23 Variants Causing Moderate to Profound Progressive Nonsyndromic Hearing Loss
Source: Genes (Basel). 2020 Dec 9;11(12):1474. doi: 10.3390/genes11121474 (PMC7764456; doi:10.3390/genes11121474)
Supplement: Supplementary file 1 [file genes-11-01474-s001.pdf]

Table S1. List of shared variants among the two affected siblings (IV-5 and IV-6, Figure 1A) after strategic filtration as depicted in Figure 2, with *CDH23* pathogenic variants highlighted.

Chromosome	Position	Reference	Altered	Gene_Transcript	cDNA	Protein	Zygosity
4	156,864,359	C	A	*CTSO*: NM_001334.2	c.193G>T	p.Ala65Ser	Heterozygous
5	140,263,953	G	C	*PCDHA13*: NM_018904.2	c.2100G>C	p.Leu700Phe	Heterozygous
7	33,059,315	C	A	*NT5C3A*: NM_001002010.2	c.477G>T	p.Leu159Phe	Heterozygous
7	95,035,508	C	T	*PON2*: NM_000305.2	c.829G>A	p.Gly277Arg	Heterozygous
7	106,826,287	A	G	*HBP1*: NM_001244262.1	c.470A>G	p.His157Arg	Heterozygous
7	142,460,374	A	G	*PRSS1*: NM_002769.4	c.547A>G	p.Met183Val	Heterozygous
9	35,819,992	A	G	*FAM221B*: NM_001012446.3	c.748T>C	p.Tyr250His	Heterozygous
10	73,464,686	G	A	*CDH23*: NM_022124.6	c.2752G>A	p.Asp918Asn	Heterozygous
10	73,537,600	T	A	*CDH23*: NM_022124.6	c.5009T>A	p.Val1670Asp	Heterozygous
12	65,232,469	A	G	*TBC1D30*: NM_015279.1	c.770A>G	p.Tyr257Cys	Heterozygous
12	81,111,002	C	A	*MYF5*: NM_005593.2	c.160C>A	p.His54Asn	Heterozygous
14	20,528,916	C	A	*OR4L1*: NM_001004717.1	c.713C>A	p.Ser238Tyr	Heterozygous
17	45,915,694	C	T	*SCRN2*: NM_138355.3	c.1061G>A	p.Arg354His	Homozygous
17	56,384,308	C	T	*BZRAP1*: NM_004758.3	c.5005G>A	p.Asp1669Asn	Heterozygous
21	43,412,301	A	G	*ZBTB21*: NM_001098402.1	c.1904T>C	p.Leu635Pro	Heterozygous
22	42,524,814	A	G	*CYP2D6*: NM_000106.5	c.638T>C	p.Leu213Pro	Heterozygous
X	118,603,873	G	T	*SLC25A5*: NM_001152.4	c.361G>T	p.Gly121Cys	Heterozygous
